# Association of Sperm Aneuploidy Frequency and DNA Fragmentation Index in Infertile Men

**Published:** 2019

**Authors:** Meenakshi Arumugam, Deyyanthody Prashanth Shetty, Jayarama Shetty Kadandale, Suchetha Kumari Nalilu

**Affiliations:** - KSHEMA Centre for Genetic Services, K. S. Hegde Medical Academy, Nitte University, Mangalore, Karnataka, India

**Keywords:** DNA fragmentation, Male infertility, Sperm aneuploidy, TUNEL assay

## Abstract

**Background:**

For improving the evaluation of male infertility, many parameters were studied and reported in earlier literature. The aim of this study was to estimate the frequency of sperm aneuploidy and DNA fragmentation in infertile men and to assess the correlation between sperm aneuploidy and DNA fragmentation.

**Methods:**

In this study 100 infertile men were included, cases with azoospermia were 68%, oligospermia 18%, severe oligospermia 6%, and oligoasthenoteratospermia (OAT) 8%. Ten normozoospermic men who had two normal children were included as a control. The sperm aneuploidy test by Fluorescence *In Situ* Hybridization (FISH) and sperm DNA fragmentation index by TdT (Terminal deoxynucleotidyl transferase)-mediated dUTP nick end labelling (TUNEL) were carried out. To determine the aneuploidy status and DNA fragmentation index, frequency was used. The correlation between sperm aneuploidy and sperm DNA fragmentation along with age was assessed by using Spearman’s correlation coefficient. The p<0.05 was considered significant.

**Results:**

The age of 100 subjects ranged between 22–48 years (35.5±5.1). Sperm aneuploidy frequency and DNA fragmentation rate were found to be higher in infertile men compared to control men (n=10). There was a significant relationship between age and sex chromosomal aneuploidy (p<0.05) and significant difference between sperm aneuploidy and damaged DNA (p<0.05).

**Conclusion:**

FISH and TUNEL assay results showed increased sperm aneuploidy frequency, and DNA fragmentation index in infertile men compared with the fertile men. There is significant relationship observed between sperm aneuploidy and DNA fragmentation. These two parameters are important and they must be investigated for clinical practice.

## Introduction

Infertility is a relatively significant problem that affects approximately 1 in 6 couples world-wide (1). Among them, male factor contributes approximately to 50% of the cases (2, 3). Wide ranges of factors are involved in male infertility, among which genetic factors play a major role in few cases. The regular semen analysis measures only the sperm production and sperm quality which can not reveal the reason for infertility even with normal semen parameters. A single definite factor influencing male infertility is the sperm DNA integrity. The possible sperm nuclear variations are abnormal chromatin structure, Y chromo-some microdeletion, sperm aneuploidy and DNA fragmentations (3). Non-disjunction and anaphase lag are the two mechanisms causing chromosome segregation in meiosis, primarily the non-disjunction leading to production of aneuploid sperm during spermatogenesis in human (4).

The common treatment for severe male factor infertility was intracytoplasmic sperm injection (ICSI) and increased incidence of sex chromosomal aneuploidy in offspring was evident by this method due to bypassing the natural selection criteria (5). During the process of chromatin remodeling, unrepaired DNA breaks were generated as a result of defective spermiogenesis, and oxidative stress could be the other mechanism for DNA damage (6). Sperm aneuploidy and sperm DNA fragmentation in infertile men is more frequent compared to the general population, which needs to be understood well (6, 7). Therefore, our main aim was to evaluate the rate of sperm aneuploidy and DNA fragmentation in infertile men to justify the inclusion of these parameters in routine practice.

## Methods

### Study population:

This study was performed on 100 infertile men with abnormal semen parameters who were referred to KSHEMA Centre for Genetic Services, K.S. Hegde Medical Academy from 2014 to 2018. Among them, cases with azoospermia were 68%, oligospermia 18%, severe oligospermia 6%, and oligoasthenoteratospermia (OAT) 8%. Ethics committee approval was obtained from the Institutional Ethics committee. Sperm aneuploidy test by Fluorescence *In Situ* Hybridization (FISH) and sperm DNA fragmentation test by TdT (Terminal deoxynucleotidyl transferase)-mediated dUTP nick end labelling (TUNEL) were carried out on semen samples of patients with oligospermia, severe oligospermia and OAT patients (32%). Among 32 infertile men, 14 (Severe oligospermia and oligospermia (64.7%) and oligoasthenoteratospermia (35.3%)) were willing to give their semen samples. Informed consent was obtained from all the participants. Normozoospermic men who had two normal children were considered and included as a control for sperm aneuploidy and sperm DNA fragmentation index.

### Processing and pretreatment of semen samples for sperm aneuploidy:

Semen samples were collected by masturbation in a sterile container after 3–5 days of sexual abstinence and left at room temperature for 30 *min*. Processing of semen sample was carried out as previously described by Bernardini et al.’s (8) protocol with modifications. 20 *ml* of Phosphate Buffer Saline (PBS) was added and centrifuged at 2000 *rpm* for 10 *min* and repeated for two more times to get the sperm alone. The supernatant was discarded, and 20 *ml* of pre-warmed hypotonic solution (0.075 *M* KCl) was added and incubated for 13 *min* at 37°*C*. After incubation, 1 *ml* of pre-cold Carnoy’s fixative was added and centrifuged at 2000 *rpm* for 10 *min*. Final fixation was done with 10 *ml* of fixative by using vortex and kept in the freezer for 30 *min*. The fixative change was given until obtaining a clear white pellet.

### Sperm aneuploidy by FISH protocol:

FISH was standardized based on Sarrate et al. (9) with slight modifications using FAST FISH prenatal enumeration probe kit (Cytocell, UK) to detect the aneuploid cells frequency of spermatozoa in infertile men. This kit includes the centromeric probes to study chromosomes X, Y, 18 (Green, Orange, and Blue, respectively) and unique sequence for chromosomes 13 and 21 (Green and Orange).

### Slide preparation:

Two drops of concentrated cell pellet were dropped onto a marked area of the slide and air dried. The slides were left at room temperature for 1 *hr*.

### Pretreatment of the slide:

The prepared slides were treated with 2x sodium saline citrate (SSC) for 5 minute*s*. Then slides were transferred into coupling jar containing DTT (25 *mM*) (Dithiothreitol) and treated for 10 *min*. The slides were placed in PBS for 2 *min* at room temperature and dehydrated with ethanol gradient of 70%, 85% and 100% for 2 *min* each and completely dried at room temperature. Then, the slides were treated with SSC (2x) again for half an hour and dehydrated with ethanol gradient of 70%, 85% and 100% for 2 *min* each.

### Co-denaturation:

Slides were dried at room temperature, and 4 *μl* of FAST FISH prenatal enumeration probe kit (Cytocell, UK) was added. They were covered with cover-slip and the coverslip was sealed with rubber cement. The slides were then placed in a Start Spin ThermoBrite plate overnight. Denaturation at 75°*C* for 5 *min* and hybridization at 37°*C* for 18 *hr* were done by the automated program in the ThermoBrite machine.

### Post-hybridization washes:

A coupling jar containing SSC (0.4x)/0.05% Triton x100 was placed in a 73±1°*C* water bath, 30 *min* before use. Rubber cement and coverslips were removed carefully and the slide with SSC (0.4x)/Triton x100 was treated for 1 *min*. Then, the slides were treated with SSC (2x)/Triton x100 for 5 *s* at room temperature. The slides were air dried and 5 *μl* of DAPI (4,6-diamidino 2-phenylindole) was applied in the target area and immediately covered with a coverslip and sealed.

### Enumeration and analysis of slides:

**Figure 1.**
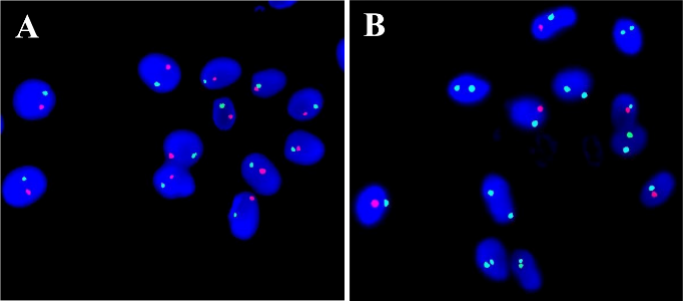
Sperm Aneuploidy by FISH. A: The picture showing the normal signal pattern of chromosome 13 (green) and 21 (red) on sperms. B: Normal signal pattern of chromosome X (green), Y (red) and 18 (Aqua) on sperm

**Figure 2.**
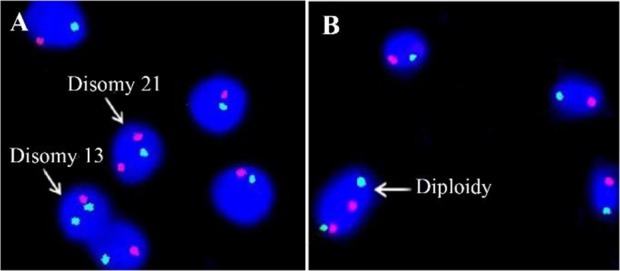
Sperm Aneuploidy by FISH. A: Sperm FISH picture showing disomy for chromosomes 13 (green) and 21 (red). B: FISH picture showing diploidy for chromosomes 13 and 21

The slides were observed using a suitable filter set on a BX 53 Olympus fluorescence microscope, and the signals present in each sperm were enumerated and captured using a CCD camera attached with the microscope. Good images were captured and documented using the GENASIS Version 7.2 Software. Sperm aneuploidy and diploidy rates for chromosome 13, 21, 18, X and Y were observed and recorded. In sperm, FISH signals were scored based on previously described criteria (2). In brief, the single signal in individual sperm represents a normal signal pattern for an exact number of chromosomes (Figures 1A and B). If additional signals were present in the cell, they were disomic for autosomes (Figure 2A) and diploid for sex chromosomes (Figure 2B). When the sperm contains two signals for each chromosome, then it is considered as diploidy. When there are no signals on sperm, it is considered as nullisomic, and nullisomic sperm could be observed in all the samples analyzed.

### DNA fragmentation index:

Terminal deoxynucleotidyl transferase-mediated deoxyuridine triphosphate (dUTP) *In Situ* DNA nick end labelling (TUNEL) assay was performed with a slight modification of sperm suspension after density gradient separation as previously described (10). A part of semen sample from the control group and patients were washed with 20 *ml* of PBS and spun at 2000 *rpm* for 10 *min*. The supernatant was discarded, and the cells were resuspended with 4% paraformaldehyde fixation buffer and permeabilized with 0.25% Triton X-100 in PBS. The supernatant was discarded and 1 *ml* of cold 70% (*v/v*) ethanol was added and stored at −20°*C* until further process. DNA strand breaks were detected by using a commercially available kit (Click-iT Plus TUNEL assay for *In Situ* apoptosis detection with Alexa Fluor dyes, molecular probes, life technologies) according to the manufacturer’s instructions.

Positive control was performed with 1 unit of DNase I diluted into DNase I Reaction Buffer (x 1) for 30 *min* at room temperature. After incubation, the slides were washed with deionized water and further proceeded to TdT reaction.

**Figure 3.**
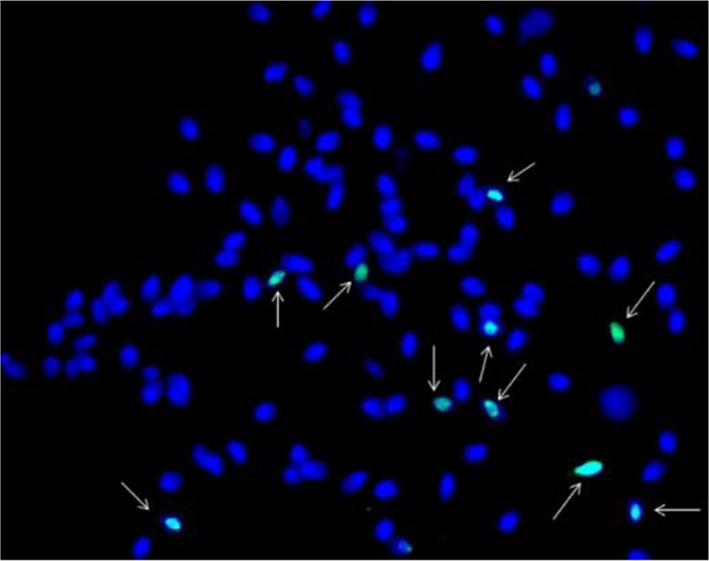
TUNEL Assay picture. Sperms with DNA damaged (green) were shown with arrows and Sperms without DNA damage were in blue color

The percentage of spermatozoa with fragmented DNA was determined by direct observation of 500 spermatozoa per sample with Olympus BX 53 fluorescence microscope. The sperm cells were counterstained with Hoechst 33342 (Blue). Fluor 488 picolyl azide dye (Green) was used to detect the sperm with TUNEL positive strand breaks. Sperm with DNA fragmentation were clearly visible in green color (Alexa), and sperm without DNA fragmentation were in blue color (Hoechst 33342) (Figure 3).

### Statistical analysis:

The collected data were summarized by using frequency/percentage for qualitative data and mean with standard deviation for quantitative data. To determine the aneuploidy status and DNA fragmentation index, frequency was used. To compare the mean of sperm aneuploidy and DNA fragmentation rate, student t- test was used. The correlation between sperm aneuploidy and sperm DNA fragmentation along with age was assessed by using Spearman’s correlation coefficient. The p<0.05 was considered statistically significant. Data management and analysis were performed by using Microsoft Excel and Statistical Package for the Social Sciences v16.0.1 (SPSS Inc, Chicago, IL, USA).

## Results

The age of 100 subjects ranged between 22–48 years with a mean and standard deviation as 35.5± 5.1. Correlation between sperm aneuploidy and DNA fragmentation with age is given in table 1. A significant correlation between age and sex chromosomal aneuploidy was observed (r=0.363, p<0.05). Sperm aneuploidy frequency (10.1±10.8) was found to be higher in infertile men as compared to fertile men. Increased disomy frequency of chromosome 13 and XY disomy was observed. The DNA fragmentation rate (61.6±21.9) was comparatively high in infertile men than fertile men. The results in table 2 indicate a positive significant difference between sperm aneuploidy and sperm with fragmented (Damaged) DNA (p<0.05); there is no significant difference between sperm aneuploidy and sperm without damaged DNA.

**Table 1.** The correlation of sperm aneuploidy and sperm DNA fragmentation with age

	**Spearman’s correlation**	**p-value**
**Autosomal aneuploidy (%)**	0.221	0.224
**Sex chromosomal aneuploidy (%)**	0.363	0.041 ^*^
**Sperm with damaged DNA (%)**	0.273	0.130
**Sperm with intact DNA (%)**	0.207	0.256

* Indicates statistical significance

**Table 2.** The obtained mean value between total sperm aneuploidy with damaged DNA and without damaged (Intact) DNA

	**Mean±SD**	**p-value**
**Sperm aneuploidy**	4.41±8.6	-
**Sperm with damaged DNA**	26.9±34.1	<0.05 ^*^
**Sperm without damaged DNA**	16.8±24.0	>0.05

* Indicates statistical significance

## Discussion

Our observation showed the percentage of sperm aneuploidy increases by the age of infertile men, mainly on sex chromosomal aneuploidy. Wyrobek et al. (11) had the largest investigation and observed the major association between age and frequencies of aneuploidy and diploid sperm. A study conducted by Martin et al. (12) observed no correlation between paternal age and “sex ratio” in sperm and aneuploidy frequency. The reason for the increased miscarriages and abnormal fetuses is abnormal sperm aneuploidy and the most widely used protocol to estimate the sperm aneuploidy frequency is FISH (13). Our results are similar to those reported by Aran et al. (14) who found an increase in sex chromosome disomy and diploid spermatozoa in infertile men. Moosani et al. (15) observed a significant increase in the frequency of disomy for chromosome 1 and XY disomy by FISH and they also observed increased frequencies of numerical abnormalities by sperm karyotyping. In our study, increased disomy frequency of chromosome 13 and XY was observed.

Our results showed an increased rate of DNA fragmentation in infertile men compared to control men, not with age. A study conducted by Singh et al. (16) revealed an increased effect of DNA damage with age and decreased age-related apoptosis in human sperm. Sperm DNA fragmentation by TUNEL assay is a good predictive parameter by its sensitivity and specificity, but not an independent measure of sperm quality (17).

The main outcome of this study indicates a positive significant correlation between sperm aneuploidy and sperm DNA fragmentation. A study conducted by Di Santo et al. (2) manifested that among 109 infertile men, a significant positive correlation existed between sperm DNA fragmentation and sperm aneuploidy and no significant correlation was found with normal men. In our study, similar results were observed. There are previous studies by different authors which confirm the same result (5–7, 18). There was a positive significant correlation between the sperm chromosome abnormality rate by FISH and DNA fragmentation even by using a combined method of Sperm Chromatin Dispersion (SCD) test (19). Another study on four infertile men who were carriers of balanced chromosomal abnormalities has shown the similar results (7). However, no significant correlation between DNA fragmentation and sperm aneuploidy was found by Balasuriya et al. (20). This type of inconsistent results explained that different tests were used to evaluate the sperm DNA fragmentation; there was a different number of probes to detect sperm aneuploidy by FISH and a different method of semen preparation.

## Conclusion

In conclusion, it can be concluded that the sperm aneuploidy and sperm DNA fragmentation are important parameters and they may be suitable for clinical practice. The reason that sperm assay is not carried out in routine practice is due to the lack of standardized protocols. By the current study, these techniques were standardized and could be used for regular diagnosis. High frequency of sperm aneuploidy and sperm DNA fragmentation might contribute to low fertilization rate and poor pregnancy outcome. There is significant relationship observed between sperm aneuploidy and DNA fragmentation. These two parameters are important and has to be investigated separately for clinical practice. However, large scale studies with specific infertile men as the subgroup may have benefit for specific therapeutic management.

## References

[1] Harton GL Tempest HG Chromosomal disorders and male infertility. Asian J Androl. 2012;14(1):32–9. 22120929 10.1038/aja.2011.66 PMC3735152

[2] Di Santo M Tarozzi N Nadalini M Borini A Analysis of sperm DNA fragmentation and aneuploidy in 109 infertile patients: are the two parameters correlated? Gynecol Obstet Case Rep. 2016;2:2.

[3] Rosalia Sa Sousa M Sperm aneuploidy and DNA integrity: a review. EMJ Repro Health. 2015;1(1): 65–73.

[4] Templado C Uroz L Estop A New insights on the origin and relevance of aneuploidy in human spermatozoa. Mol Hum Reprod. 2013;19(10):634–43. 23720770 10.1093/molehr/gat039

[5] Carrell DT Emery BR Wilcox AL Campbell B Erickson L Hatasaka HH Sperm chromosome aneuploidy as related to male factor infertility and some ultrastructure defects. Arch Androl. 2004;50 (3):181–5. 15204685 10.1080/01485010490425188

[6] Muriel L Goyanes V Segrelles E Gosalvez J Alvarez J Fernandez JL Increased aneuploidy rate in sperm with fragmented DNA as determined by the sperm chromatin dispersion (SCD) test and FISH analysis. J Androl. 2007;28(1):38–49. 16899813 10.2164/jandrol.106.000067

[7] Perrin A Louanjli N Ziane Y Louanjli T Le Roy C Gueganic N Study of aneuploidy and DNA fragmentation in gametes of patients with severe teratozoospermia. Reprod Biomed Online. 2011;22 (2):148–54. 21233018 10.1016/j.rbmo.2010.10.006

[8] Bernardini L Borini A Preti S Conte N Flamigni C Capitanio GL Study of aneuploidy in normal and abnormal germ cells from semen of fertile and infertile men. Hum Reprod. 1998;13(12):3406–13. 9886524 10.1093/humrep/13.12.3406

[9] Sarrate Z Vidal F Blanco J Role of sperm fluorescent in situ hybridization studies in infertile patients: indications, study approach, and clinical relevance. Fertil Steril. 2010;93(6):1892–902. 19254793 10.1016/j.fertnstert.2008.12.139

[10] Borini A Tarozzi N Bizzaro D Bonu MA Fava L Flamigni C Sperm DNA fragmentation: paternal effect on early post-implantation embryo development in ART. Hum Reprod. 2006;21(11): 2876–81. 16793992 10.1093/humrep/del251

[11] Wyrobek AJ Eskenazi B Young S Arnheim N Tiemann-Boege I Jabs EW Advancing age has differential effects on DNA damage, chromatin integrity, gene mutations, and aneuploidies in sperm. Proc Natl Acad Sci USA. 2006;103(25): 9601–6. 16766665 10.1073/pnas.0506468103 PMC1480453

[12] Martin RH Spriggs E KO E Rademaker AW The relationship between paternal age, sex ratios, and aneuploidy frequencies in human sperm, as assessed by multicolor FISH. Am J Hum Genet. 1995;57(6):1395–9. 8533769 PMC1801415

[13] Ramasamy R Scovell JM Kovac JR Cook PJ Lamb DJ Lipshultz LI Fluorescence in situ hybridization detects increased sperm aneuploidy in men with recurrent pregnancy loss. Fertil Steril. 2015;103(4):906–9. 25707335 10.1016/j.fertnstert.2015.01.029 PMC4385482

[14] Aran B Blanco J Vidal F Vendrell JM Egozcue S Barri PN Screening for abnormalities of chromosomes X, Y, and 18 and for diploidy in spermatozoa from infertile men participating in an in vitro fertilization intra cytoplasmic sperm injection program. Fertil Steril. 1999;72(4):696–701. 10521113 10.1016/s0015-0282(99)00307-6

[15] Moosani N Pattinson HA Carter MD Cox DM Rademaker AW Martin RH Chromosomal analysis of sperm from men with idiopathic infertility using sperm karyotyping and fluorescence in situ hybridization. Fertil Steril. 1995;64(4):811–7. 7672155 10.1016/s0015-0282(16)57859-5

[16] Singh NP Muller CH Berger RE Effects of age on DNA double-strand breaks and apoptosis in human sperm. Fertil Steril. 2003;80(6):1420–30. 14667878 10.1016/j.fertnstert.2003.04.002

[17] Sergerie M Laforest G Bujan L Bissonnette F Bleau G Sperm DNA fragmentation: threshold value in male fertility. Hum Reprod. 2005;20(12): 3446–51. 16085665 10.1093/humrep/dei231

[18] Brahem S Mehdi M Elghezal H Saad A Study of aneuploidy rate and sperm DNA fragmentation in large-headed, multiple-tailed spermatozoa. Andrologia. 2012;44(2):130–5. 10.1111/j.1439-0272.2010.01115.x 21714801

[19] Enciso M Alfarawati S Wells D Increased numbers of DNA-damaged spermatozoa in samples presenting an elevated rate of numerical chromosome abnormalities. Hum Reprod. 2013;28(6): 1707–15. 23526303 10.1093/humrep/det077

[20] Balasuriya A Speyer B Serhal P Doshi A Harper JC Sperm chromatin dispersion test in the assessment of DNA fragmentation and aneuploidy in human spermatozoa. Reprod Biomed Online. 2011; 22(5):428–36. 21397561 10.1016/j.rbmo.2011.01.012

